# Fluid Resuscitation in Pediatric Cerebral Malaria: Insights from the FEAST Trial and Further Evidence from Malawi

**DOI:** 10.4269/ajtmh.25-0132

**Published:** 2025-04-01

**Authors:** Elizabeth C. George, Kathryn Maitland

**Affiliations:** ^1^ Medical Research Council Clinical Trials Unit (MRC CTU), University College London, United Kingdom;; ^2^ Institute of Global Health Innovation, Department of Surgery and Cancer, Imperial College, London, United Kingdom

Worldwide, fluid boluses remain the standard of care for the emergency treatment of shock. Unlike the situation in adults, in whom shock is defined by hypotension, pediatric shock criteria largely focus on signs of impaired perfusion,^1^^,^^2^ since it is widely held by experts informing resuscitation guidelines that hypotension is a late complication of shock, despite minimal data to support this assertion. The term “compensated shock” is therefore more widely accepted in pediatric practice and is synonymous with severely impaired perfusion. The Fluid Expansion as a Supportive Therapy (FEAST) trial was conducted to generate the relevant evidence for fluid resuscitation in resource-poor hospitals in Africa, where access to mechanical ventilation and inotropic therapy were not, and are still not, routinely available.^3^

The design of the FEAST trial was pragmatic, including large subgroups of sepsis and malaria. Overall, in the stratum with compensated shock, 57% of children had malaria parasitemia, 15% had coma, and 12% had culture-proven bacterial sepsis. The trial was stopped early by the data and safety monitoring committee due to excess mortality in children randomized to a fluid bolus compared to controls (relative risk 1.45 [1.13–1.86] *P* = 0.003).^3^ Results were consistent across subgroups, including malaria, coma, sepsis, acidosis, and severe anemia. Adverse events of suspected raised intracranial pressure and pulmonary edema were prospectively monitored and adjudicated by an endpoint committee who were blind to the intervention arm. Suspected increased intracranial pressure events occurred in 2.6%, 2.2%, and 1.7% (*P* = 0.17) in the albumin-bolus, saline-bolus and control (no bolus) arms, respectively.

In this issue of the *American Journal of Tropical Medicine and Hygiene,* Sherman et al. report on a retrospective analysis of fluid bolus administration in 1674 children with cerebral malaria enrolled into research studies in Blantyre, Malawi from 2000 to 2018.^4^ They used methodology that allowed observational data to mimic some of the characteristics of a randomized trial, through calculating a propensity score – a probability that a participant receives an intervention or treatment conditional on their baseline characteristics. That score is used to balance between groups receiving and not receiving an intervention, attempting to create the balance of baseline characteristics achieved through randomization in a clinical trial. Their findings support the results of FEAST, with increased mortality in the fluid-bolus group (OR 1.92; 95% CI: 1.36–2.71) compared to those who did not receive a bolus. In a supplemental file, the authors report the proportion of children with cerebral malaria who received a fluid bolus before and after publication of the FEAST trial; use of fluid boluses decreased from 16.3% to 12.1%. Although we calculated that this reduction was statistically significant (*P* = 0.05), it is disappointing that administration of fluid boluses remains common, and we hope that the data presented in the current report will further discourage fluid bolus use in children with cerebral malaria, since this intervention nearly doubled their risk of dying.

We note, that in the discussion, the authors referenced only one of the two secondary analyses of the FEAST trial seeking to understand modes of death. The first, using competing risk analyses to estimate cumulative incidence curves by mode of death and to thereby estimate sub-hazard ratios comparing randomized arms, was not considered by the authors. Those analyses demonstrated that excess deaths were due to cardiovascular collapse (*n* = 123 deaths; 4.6% versus 2.6%, *P* = 0.008), whereas number of deaths due to suspected pulmonary edema (*n* = 61 deaths; 2.2% versus 1.3%, *P* = 0.09) and neurological causes (*n* = 63, 2.1% versus 1.8%, *P* = 0.6) did not differ significantly between the groups.^5^ The second analysis, which was highlighted in the discussion, included the creation of composite “physiological” scores (categorizing children into respiratory, neurological, and cardiovascular phenotypic risks) as baseline predictors of mortality.^6^ Quartango et al. pointed out that incorrect statistical methods were used for the analyses, questioning the validity of the findings.^7^ Furthermore, the study included 100% imputation of biochemistry values at one hour and use of a multiplication factor in the bolus arms to derive the scores, leading to misleading conclusions due to confounding bias within the treatment assignments (since the multiplication factor did not apply to the non-bolus arm). Even if the methods were statistically sound, the differences seen in the derived clinical scores between arms by no means implies causality with respect to mode of death.^7^ Nevertheless, this study is frequently cited as a justification to provide fluid boluses alongside respiratory support in children with shock.^8^

In conclusion, we highlight to pediatricians across Africa managing critically ill children that in 2020, the Pediatric Surviving Sepsis Campaign guidelines were updated. They indicate that, “in healthcare systems with no availability of intensive care and in the absence of hypotension, *we recommend against bolus fluid administration* while starting maintenance fluids (strong recommendation, high quality of evidence)”.^9^ The FEAST trial showed harm for a fluid bolus in every subgroup (including those with sepsis or malaria) and for every definition of shock (except severe hypotension, for which there was no control group).^10^ Given the most common cause of coma in critically ill children in malaria-endemic sub-Saharan Africa is cerebral malaria, by implication, the FEAST results extend to this group. The analysis presented in this issue, in a routine healthcare setting in Malawi, also supports the conclusion that fluid boluses are not indicated for children with cerebral malaria.
